# Pathogenesis of pulmonary hypertension caused by left heart disease

**DOI:** 10.3389/fcvm.2023.1079142

**Published:** 2023-03-03

**Authors:** Mingzhu Xiao, Disheng Lai, Yumin Yu, Qingqing Wu, Caojin Zhang

**Affiliations:** ^1^Guangdong Provincial Key Laboratory of Pharmaceutical Bioactive Substances, School of Traditional Chinese Medicine, Guangdong Pharmaceutical University, Guangzhou, Guangdong, China; ^2^Department of Cardiology, Guangdong Provincial People’s Hospital (Guangdong Academy of Medical Sciences), Southern Medical University, Guangzhou, Guangdong, China

**Keywords:** pulmonary hypertension, vasoconstriction, vasodilatation, vascular remodeling, mitochondria, ion channel, inflammatory cytokines

## Abstract

Pulmonary hypertension has high disability and mortality rates. Among them, pulmonary hypertension caused by left heart disease (PH-LHD) is the most common type. According to the 2022 ESC/ERS Guidelines for the diagnosis and treatment of pulmonary hypertension, PH-LHD is classified as group 2 pulmonary hypertension. PH-LHD belongs to postcapillary pulmonary hypertension, which is distinguished from other types of pulmonary hypertension because of its elevated pulmonary artery wedge pressure. PH-LHD includes PH due to systolic or diastolic left ventricular dysfunction, mitral or aortic valve disease and congenital left heart disease. The primary strategy in managing PH-LHD is optimizing treatment of the underlying cardiac disease. Recent clinical studies have found that mechanical unloading of left ventricle by an implantable non-pulsatile left ventricular assist device with continuous flow properties can reverse pulmonary hypertension in patients with heart failure. However, the specific therapies for PH in LHD have not yet been identified. Treatments that specifically target PH in LHD could slow its progression and potentially improve disease severity, leading to far better clinical outcomes. Therefore, exploring the current research on the pathogenesis of PH-LHD is important. This paper summarizes and classifies the research articles on the pathogenesis of PH-LHD to provide references for the mechanism research and clinical treatment of PH-LHD, particularly molecular targeted therapy.

## Introduction

Pulmonary hypertension (PH) represents a grave condition associated with high morbidity and mortality (1). PH is a clinical and pathophysiological syndrome of elevated pulmonary vascular resistance and pulmonary artery pressure caused by structural or functional changes in the pulmonary vasculature caused by multiple heterogenous diseases. Among them, pulmonary hypertension caused by left heart disease (PH-LHD) is the most common type (2). PH is defined by a mean pulmonary arterial pressure (mPAP) >20 mmHg. Consistent with the general definitions of PH, PH-LHD is defined by a mPAP >20 mmHg and a pulmonary artery wedge pressure (PAWP) >15 mmHg. PH-LHD can be divided into 2 subcategories, defined as isolated post-capillary pulmonary hypertension (IpcPH) and combined post- and pre-capillary pulmonary hypertension (CpcPH). Within this hemodynamic condition of post-capillary PH, IpcPH is defined by Pulmonary vascular resistance (PVR) ≤ 2 WU and CpcPH by PVR > 2 WU (3). PH-LHD belongs to postcapillary pulmonary hypertension, which is distinguished from other types of pulmonary hypertension because of its elevated PAWP. According to the 2022 ESC/ERS Guidelines for the diagnosis and treatment of pulmonary hypertension, PH-LHD is classified as the second major category, which is left heart disease associated pulmonary hypertension. PH-LHD accounts for approximately 65–80% of all patients with PH (4). PH-LHD includes PH caused by mitral or aortic valve disease, left ventricular dysfunction, and congenital left heart disease (5). In recent years, many explorations have been made in the treatment of PH-LHD, but still no breakthrough has been achieved (6). In addition, current clinical treatment is still based on symptomatic and supportive treatment because the pathogenesis remains unclear. At present, PH-LHD is difficult to prevent and cure after the disease, for example, pulmonary hypertension caused by congenital cardiovascular conditions is classified as a congenital disease, which cannot be prevented and avoided to the extent of recurrence, and it requires extensive medication to maintain the prognosis. Based on the results of clinical studies, current targeted drug therapy is not suitable for widespread use in the treatment of patients with PH-LHD. Therefore, identifying the exact pathogenesis and therapeutic target of PH-LHD can promote the administration of targeted therapy, thereby increasing the cure rate.

In this paper, we summarized the research articles on the pathogenesis of PH-LHD, which were then categorized and reviewed. On this basis, we proposed new ideas to provide a reference for the study of the mechanism of PH-LHD and clinical treatment, particularly molecular targeted therapy. PH-LHD was once considered to be a disease dominated by increased vascular tension and thrombosis, and is now considered to be a vasculopathy. The related mechanism of vascular cells plays a major role in the occurrence and development of the disease. The etiology of PH-LHD is primarily due to a passive increase in pulmonary vein resistance, which is usually accompanied by an increase in pulmonary vascular tone (vasoconstriction) and extensive pulmonary vascular remodeling. Therefore, this paper focuses on the pathogenesis of pulmonary vasoconstriction and pulmonary vascular remodeling mediated by vascular cells.

### Pathobiology of pulmonary hypertension related to left heart disease

PH-LHD patients includes patients with heart failure (HF) with reduced, mildly reduced, or preserved ejection fraction (HFrEF, HFmrEF, or HFpEF), left-sided valvular heart disease, and congenital/acquired cardiovascular conditions leading to post-capillary PH. The pathophysiological processes that lead to all these forms of PH-LHD patients are as follows: (1) An initial passive increase in left ventricular (LV) filling pressures and backward transmission into the pulmonary circulation. LV systolic and diastolic dysfunction and aortic and/or mitral stenosis and/or regurgitation can increase left ventricular filling pressure, followed by elevated left atrial pressure (LAP) for a period of time. LAP can then be transmitted backwards through the pulmonary vein to the pulmonary vascular system, resulting in pulmonary artery intima thickening and media hypertrophy and PH (7). (2) Endothelial dysfunction (including vasoconstriction). When left atrial pressure increases, two major vascular changes occur. The first is stress failure of capillaries and alveolar membranes, manifested as a typical acute phenomenon caused by barotrauma of pulmonary microvessels, which destroys endothelial function and permeability and impairs the biological and functional properties of alveolar units. Another phenomenon is the process of vascular remodeling, which is related to sustained pressure injury and involves capillaries, especially small arterial walls (8). (3) Vascular remodeling (which may occur in both venules and/or arterioles). The development of PH in left heart failure is not only caused by the passive increase of pulmonary vascular pressure, but also usually aggravated by the concomitant increase of PVR, which can be attributed to increased pulmonary vascular tension (9) and extensive pulmonary vascular remodeling (10). Both phenomena are thought to be caused by pulmonary endothelial dysfunction, characterized by an imbalance between vasodilation and vasoconstriction factor secretion (11). (4) RV dilatation/dysfunction. PH leads to increased right ventricular load and pressure, resulting in right ventricular (RV) dilation. Right ventricular remodeling from crescent to spherical leads to functional tricuspid regurgitation and elevated right atrial pressure (12). (5) Altered RV-PA coupling. RV-pulmonary artery (PA) coupling refers to the relationship between RV contractility and RV afterload. Contraction force is an inherent RV function. RV and PA are “coupled” because RV contraction should “match” the afterload. If RV afterload increases, RV contractility should also increase (i.e., through RV hypertrophy and load adaptation) in order to maintain RV function and RV-PA coupling (13). The compensatory mechanism of RV to pulmonary hypertension is hypertrophy. RV dilation, oxygen supply and demand imbalance and RV ischemia occur, resulting in decreased contractility, RV can not maintain cardiac output, inevitably “uncoupling,” compensatory disorders begin (7).

### Distinction between IpcPH and CpcPH

Pre-capillary PH is caused by remodeling of pulmonary arteries and small arteries and PAH is a pre-capillary PH. Both IpcPH and CpcPH are characterized by elevated mPAP and PAWP. In most patients with IpcPH, elevated mPAP can be attributed to elevated left ventricular filling pressure, but it may still be caused by pulmonary vascular remodeling (14). The increase of mPAP in CpcPH patients is disproportionate to the pressure generated by left ventricular filling pressure conduction, and the increase of mPAP is usually more serious than that in IpcPH patients. Patients in this group developed pulmonary vascular disease that can be attributed to chronic vasoconstriction and pulmonary vascular remodeling (15). CpcPH also exhibits hypertrophic remodeling, fibrosis, and luminal occlusion of the distal pulmonary artery (16). CpcPH is hemodynamically more similar to PAH than IpcPH, (17) and it may have similar vascular remodeling as PAH (18).

IpcPH, also known as passive PH, is PH caused by a passive increase in LAP transmitted backward through the pulmonary veins to the pulmonary vascular system, regardless of the presence of underlying valvular disease, HFrEF or HFpEF (19). The increase in PVR and mPAP in Ipc-PH was driven solely by elevated LAP (20). This level of vascular tone is regulated by the endothelial and vascular smooth muscle layers and the interlayer interactions, which respond to different stimuli, such as local metabolic regulation, and endothelial products (21). Over time, pulmonary vein hypertension and potential LHD disease can further trigger vasoconstriction and vascular remodeling of the pulmonary precapillary components. Histologically, intimal and medial hypertrophy of the pulmonary artery was observed, which led to pathological obstruction of the distal pulmonary artery and subsequent significant increase in PVR, which was called CpcPH (19). There is growing evidence that CpcPH patients have different pathophysiology (22). The pathogenic mechanisms of IpcPH and CpcPH are not exactly the same, so the distinction between IpcPH and CpcPH is crucial for treatment selection.

### Multiple factors stimulate vasoconstriction leading to PH-LHD

The narrowing of the vascular lumen caused by increased vascular tone is called vasoconstriction. Vasoconstriction is a characteristic feature of pulmonary hypertension. Ventricular diastole, systolic dysfunction, and valvular disease can induce PH-LHD, which primarily leads to increased left heart filling pressure, obstruction of pulmonary venous return, pulmonary vasoconstriction, increased pulmonary venous pressure, and increased pulmonary arterial pressure. Sustained increases in cardiac filling and pulmonary venous pressures may lead to superimposed effects on the pulmonary circulation, including decreased nitric oxide (NO) utilization, increased endothelin-1 (ET-1), and infiltration of metabolic factors, which cause sustained pulmonary vasoconstriction (23). Endothelial cell (EC) dysfunction plays a decisive role in the pathogenesis of PH-LHD. It also leads to disturbance of vasoactive mediators and imbalance of contractile and diastolic factors, primarily involving three pathways, including ET-1, prostacyclin (PGI2), and phosphodiesterases (PDEs). Apart from the three pathways, 5-hydroxytryptamine (5-HT) and ion channels are also involved in this process (24).

### ET-1 pathway

**Figure 1:**
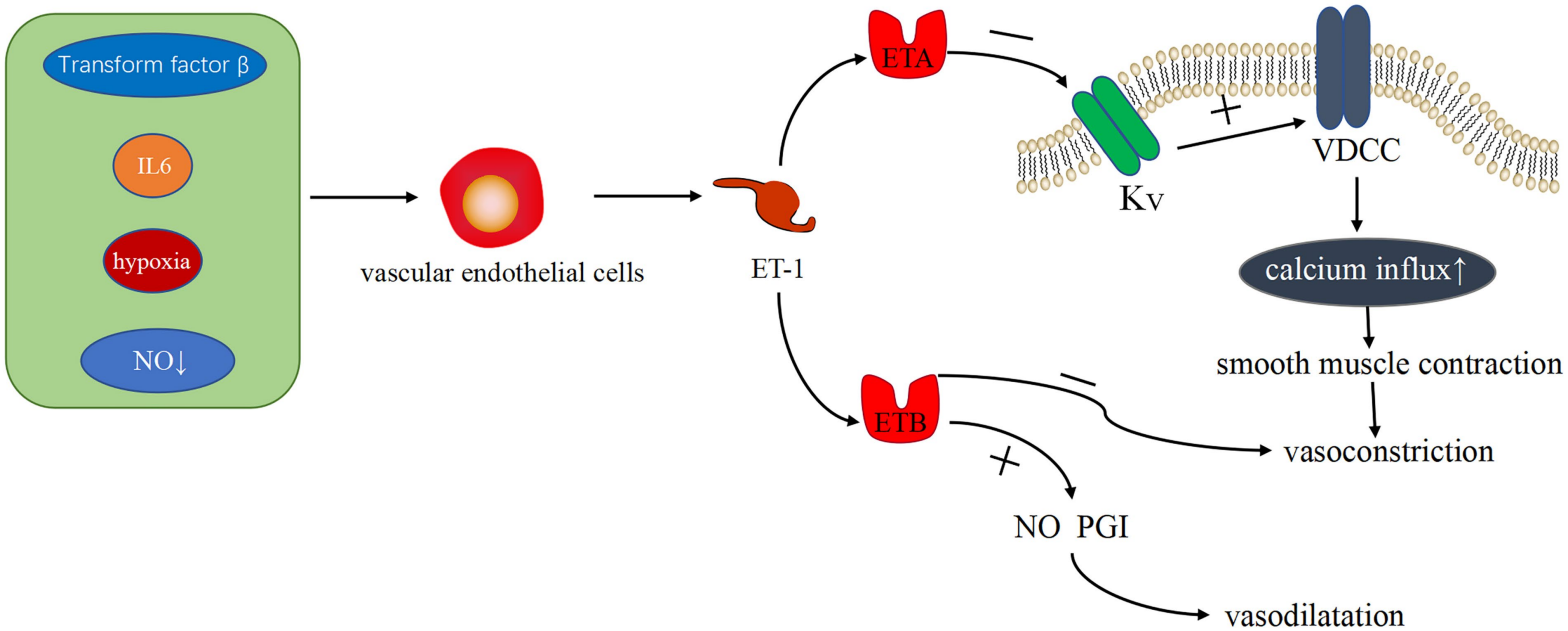
Related pathways of ET-1 vasoconstriction. Transforming factor-β, IL-6, hypoxia, and NO reduction can stimulate endothelial cells to produce ET-1. The binding of ET-1 and ET-1A receptor protein can inhibit the K^+^ channel, activate Ca^2+^ channel, increase Ca^2+^ influx, and cause vasoconstriction. ET-1 binding to ET-1B receptor protein can clear ET-1, inhibit vasoconstriction, and promote the production of vasodilator substances, such as NO and PGI, causing vasodilation.

ET-1 is the most effective endogenous vasoconstrictor known to date. Vasoconstriction induces the proliferation of pulmonary artery smooth muscle cells (PASMCs), in which ET-1 serves as a vasoconstrictor. Several studies (25, 26) have shown that ET-1 is produced by vascular ECs and acts by binding to two two different protein-coupled receptors, namely, ETA and ETB. In the cardiovascular system, ETA is primarily expressed on vascular smooth muscle cells (VSMC), where it mediates smooth muscle proliferation, leading to vasoconstriction. On the contrary, ETB is involved in scavenging endothelin and promoting the production of vasodilators (e.g., NO and PGI), which is primarily expressed on VSMC and ECs (27). Veteskova et al. found elevated levels of ET-1 in patients with PH-LHD (28), ET-1 activates Ca^2+^ channels in the cell membrane by working to activate ETA to inhibit K^+^ channels causing increased calcium inward flow and increased Ca^2+^ concentration, resulting in vasoconstriction and proliferation of PASMCs (29) (Figure 1). Hypertrophic proliferation of PASMCs increases the level of ET-1, thereby causing imbalance in the level of ET-1 clearance and secretion and exhibiting persistent constriction in blood vessels. Such inflammatory factors, including interleukin-6 (IL-6), transforming factor-β (30, 31), increased reactive oxygen species in the pulmonary vasculature (32), and lack of NO (28) and O_2_, can stimulate ET-1 production by ECs and exacerbate vasoconstriction. Notably, ET-1 has a strong vasoconstrictive effect, and it can be produced through multiple pathways, which plays an important role in vasoconstriction.

### Phosphodiesterases

**Figure 2:**
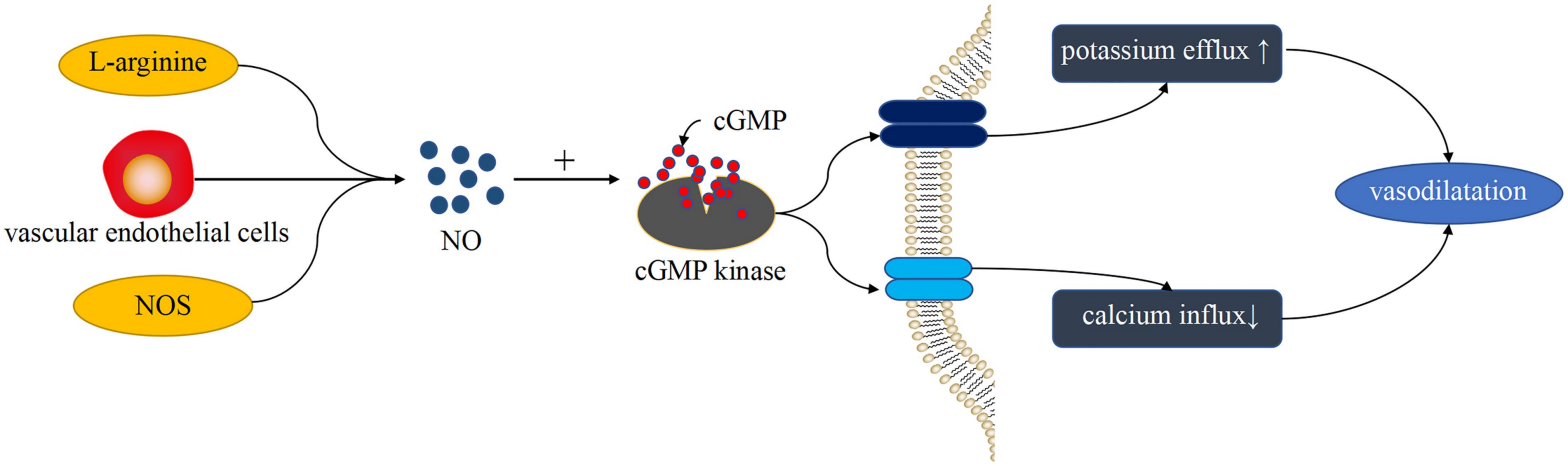
Vasodilation-induced pathways of NO. l-arginine generates NO under the catalysis of NOS. NO production is also affected by ET-1 produced by endothelial cells. NO plays a role through the cGMP-cGMP kinase pathway, which causes K^+^ and Ca^2+^ transport imbalance, resulting in vasodilation.

**Figure 3:**
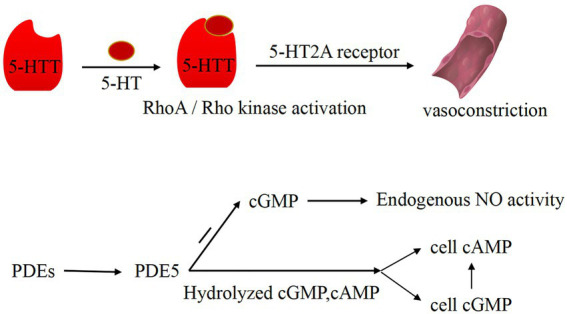
Vasoconstriction pathways induced by PDEs and 5-HT. 5-HT and 5-HT transporter (5-HTT) are involved in the activation of the RhoA/Rho kinase pathway in phosphate smooth muscle cells. Under the action of 5-HTT and RhoA/Rho kinase, 5-HT binds to 5-HT2A receptor and induces vasoconstriction. PDE5 can degrade GMP, thereby reducing the activity of endogenous NO. In addition, PDE5 can hydrolyze intracellular cAMP and cGMP to regulate cAMP level through the high level of cGMP.

PDEs are a diverse superfamily of enzymes that regulate the cellular levels of the secondary messenger molecules cAMP and cGMP by catalyzing the hydrolysis of their phosphodiester bonds. Intracellular cAMP and cGMP play a key role in cell signaling by activating protein kinases, while NO, a cell signaling molecule with a regulatory role in vascular tone, also induces the production of cGMP (33). Ntontsi et al. showed that the superfamily of PDEs regulate the levels of cellular cAMP and cGMP and that changes in their levels can cause vasoconstriction (34). PDE is widely distributed in the body, and it works by hydrolyzing cAMP and cGMP in cells to regulate the level of cAMP through high levels of cGMP. Mokry et al. found that PDE5 plays a major role in the pulmonary vasculature (35) and that PDE5 decreases the activity of the endothelium-derived diastolic factor NO by degrading cGMP (36). In addition, NO plays a partially important role in counteracting the increase in pulmonary artery tone (37), which is produced by L-arginine catalyzed by nitric oxide synthase (NOS). Based on related studies, endogenous NO production is also influenced by ET-1, and they interact with each other. Klinger et al. suggested that NO plays an important role in vasodilation and inhibition of PASMC proliferation and migration (38). All three isoforms of NOS regulate pulmonary vascular tone, and they are expressed in the lung (5). Vanhoutte et al. showed that NO in vascular smooth muscle acts through the cyclic guanosine monophosphate (cGMP)-protein kinase G pathway (39). As a second messenger, cGMP activates cGMP kinase to activate K^+^ channels in the membrane, causing an imbalance in K^+^ and Ca^2+^ transport and vasodilation (Figure 2). That is, NO mediates this process by increasing cGMP products. Therefore, PDE can block the NO/cGMP pathway by degrading cGMP and causing an imbalance in systolic–diastolic levels, resulting in vasoconstriction (Figure 3). Therefore, overexpressed PDEs serve as markers of vasoconstriction.

### PGI2 pathway

**Figure 4:**
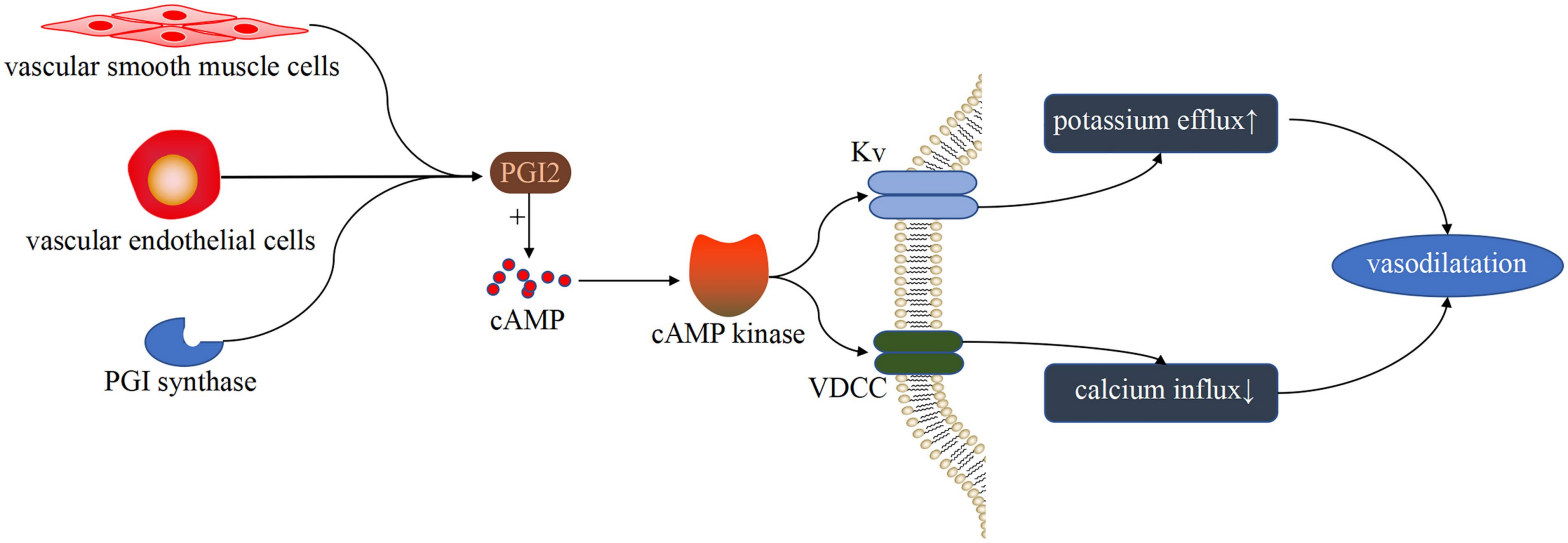
Vasodilatation pathway induced by PGI. Under the catalysis of PGI synthase, endothelial cells and vascular smooth muscle cells produce PGI. After binding to G protein-coupled receptors, PGI activates the cAMP pathway and inhibits the activity of VDCC and KV, resulting in a decrease in K^+^ efflux and Ca^+^ influx, as well as vasodilation.

PGI2 is primarily produced by endothelial and vascular smooth muscle cells, and it stimulates pulmonary artery vasodilation and inhibits vascular smooth muscle cell proliferation by increasing cAMP through activation of the corresponding receptors (40). PGI2 can also enhance NO production *via* endothelial cells (41). Moncada et al. pioneered the discovery that PGI2 can cause pulmonary vasodilation and inhibit smooth muscle growth (42). Some studies have shown that G protein-coupled receptor activation of the cAMP pathway is involved in PGI2-induced vasoconstriction (43) (Figure 4). In addition, Rucker et al. showed that PGI and thromboxane are involved in maintaining vascular autostasis (44, 45). Reduced PGI levels and increased thromboxane levels were found in constricted pulmonary arteries of patients with PH-LHD.

### 5-HT

5-HT is synthesized by the pulmonary artery endothelium and can act on PASMC in a paracrine manner. 5-HT enters PASMC cells *via* serotonin transporter protein (SERT) and activates PASMC contractile and proliferative signaling pathways by binding to receptors on the plasma membrane (46). The vasoconstrictor 5-HT interacts with the receptor, resulting in abnormal constriction of pulmonary arteries and abnormal proliferation of PASMCs and narrowing the vascular lumen vascular resistance, thereby leading to PH-LHD (47). 5-HT and the 5-HT transporter (5-HTT) are involved in activation of the RhoA/Rho kinase pathway in phospho-smooth muscle cells (48). 5-HT promitogenesis, which is mediated by 5-HTT and RhoA/Rho kinase activation, induces the proliferation of PASMCs, thereby causing vasoconstriction (Figure 3). Among the 14 5-HT receptor subtypes, 5-HT1B and 5-HT2A receptors were found to be involved in mediating pulmonary vasoconstriction, and 5-HT-induced vasoconstriction was primarily dependent on 5-HTT and 5-HT2A receptors.

### Calcium channels

Increased concentration of free Ca^2+^ in PASMC cytoplasm can trigger vasoconstriction and also play a key role in stimulating PASMC proliferation and migration (49). The increase of intracellular calcium ions is the main cause of vasoconstriction caused by multiple classes of pathways; therefore, Ca^2+^ channels may also be involved in PH-LHD processes. Breitling et al. (50) showed that excitation–contraction coupling of pulmonary vascular smooth muscle depends on Ca^2+^ signaling regulation, that is, VDCC on the membrane of PASMCs is induced by ET-1, causing Ca^2+^ inward flow and Ca^2+^ release from IICR and CICR in PASMCs (5) and promoting smooth muscle contraction, which eventually manifests as pulmonary vasoconstriction. Therefore, calcium channels are important for the formation of vasoconstriction.

### Calcium-activated chloride channels

**Figure 5:**
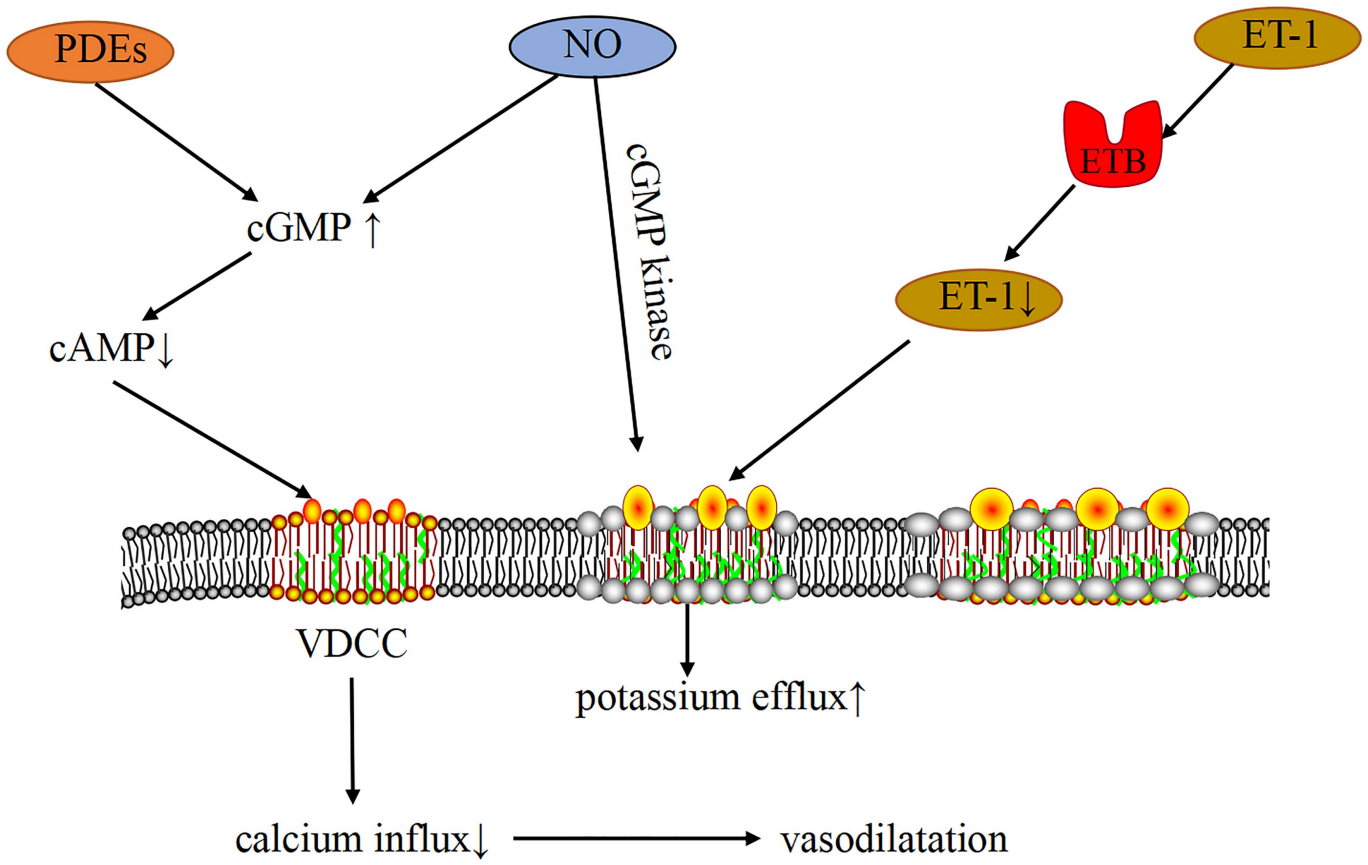
Mechanism of PH-LHD-related vasoconstriction induced by the ion channel. PDEs and NO can increase cGMP, decrease cAMP, inhibit the activity of VDCC, reduce Ca^2+^ influx, and cause vasodilation. In addition, cGMP can activate KV through cGMP kinase, thereby inhibiting the activity of VDCC and causing vasodilation. ET-1 binds to ET-1B receptor, which can clear ET-1, decrease ET-1 concentration, activate KV, inhibit VDCC activity, increase Ca^2+^ influx, and cause vasodilation.

Chloride channel currents are triggered by an increase in intracellular Ca^2+^ concentration, which is known as calcium-activated chloride channels (CaCC). In vascular smooth muscle tissue, CaCC provide the major anion channels by opening these channels to allow chloride efflux and depolarization of the cell membrane. Simultaneously an increase in intracellular [Ca^2+^] also activates the channels, producing an increase in excitability, initiating action potentials and contraction or increasing tension (51). A related study (52) pointed out that chloride calcium channel density is increased in patients with pulmonary hypertension, and chloride calcium channels mediate chloride outflow and reduce intracellular membrane negative charge, resulting in the depolarization of PASMCs, further Ca^2+^ inward flow, activation of the calcium regulatory protein system, and vasoconstriction. In addition, another ion channel, namely, the volume-sensitive chloride channel (Clswell) (5), has a vasoconstrictive effect on blood vessels when osmotic pressure decreases or vascular wall tension increases. Calcium activates chloride channels, which together with Ca^2+^ have a constrictive effect on the vasculature. Three major pathways, including ET-1, NO, and PGI2, produce vasoconstrictive effects through two pathways, namely, cAMP and cGMP. Vasoactive mediators (including PDEs) inhibit potassium channels, which in turn activate calcium channels, causing increased calcium inward flow and sustained vasoconstriction (Figure 5). 5-HT binds to receptors and acts directly on vascular smooth muscle cells. In general, vasoconstriction is only the initial manifestation, and sustained vasoconstriction also causes vascular remodeling, but detecting pathophysiological changes in the vasculature during the initial stage is difficult. Therefore, PH-LHD is difficult to diagnose in the early stages.

### Multiple factors lead to vascular remodeling that triggers PH-LHD

Vascular remodeling is the corresponding change of vascular wall to adapt to the chronic increase of intravascular pressure: the vessel wall thickens, decreases in lumen diameter, reduces vasodilation capacity, and subsequently increases pulmonary vascular resistance and sustained PH (50).

Pulmonary artery remodeling (PAR) is a main characteristic of all forms of pulmonary hypertension and a key factor in its pathogenesis (53). Characteristics of pulmonary blood vessel remodeling include thickening and strengthening of the intimal and/or medial muscular vessel layers. Left ventricular systolic and diastolic dysfunction and heart valve disease will increase the filling pressure of the left ventricle, and the pressure will increase in the subsequent period of time. Given the extensive structural changes in the pulmonary vascular wall, the changes observed in PH-LHD arise secondary to a sustained increase in intravascular pressure. Left atrial pressure can be transmitted back to the pulmonary vascular system through the pulmonary vein, thereby affecting the pulmonary vascular system. Vascular remodeling begins as an adaptation to the chronic increase in intraluminal pressure. As a result of vascular remodeling, the condition began to deteriorate continuously, and transpulmonary pressure gradient and pulmonary vascular resistance increased, whereas the efficacy of drugs decreased or showed no response, which is known as no vascular reactivity or refractory type. Avascular reactivity results from PAR, which makes pulmonary vessels stiff and less reactive to vasodilators. Vascular remodeling is by far an important and intractable factor. Therefore, vascular remodeling remains an important factor in pulmonary hypertension, which is defined as a chronic incurable disease (54).

With regard to the etiology and mechanism of vascular remodeling, the currently studied mechanisms include genetic inheritance, mast cell accumulation, inflammatory factors, and mitochondrial dysfunction.

### Genetic inheritance

Most of current knowledge in this area comes again from the field of PAH. Lars C. Myung-Jin Kim and other scholars (55) believed that BMPR2 can be expressed on pulmonary endothelial and vascular smooth muscle cells. Upon binding of specific ligands (particularly BMP-2 and BMP-4), BMPR2 heterodimerizes with BMPR1, resulting in the activation and inhibition of differentiation proteins. These downstream signaling events activate master regulators of cell cycle control, inhibit proliferation, activate apoptosis, and induce cell senescence. Evidence suggests that dysfunctional BMPR2 results in the imbalance of proliferation and apoptosis, thereby leading to vascular remodeling. Other related studies (56, 57) have also suggested that BMPR2 plays an important role in regulating vascular proliferation. BMPR2 mutations are the most common inherited risk factors for PAH. The KCNK3 mutations are also a inherited risk factors for PAH (58). KCNK3 regulates the resting membrane potential in several cell types, including PASMCs and RV cardiomyocytes, in which its protein is an outward K+ channel (59). A recent study showed that KCNK3 channel dysfunction also plays a key role in PH-LHD (60). The study suggests that Kcnk3-LOF (loss-of-function) mutations promote the destruction of pulmonary endothelial integrity in the event of LV pressure overload, leading to perivascular edema and PA remodeling. Meanwhile, inflammatory signal (IL-6) leads to PASMC proliferation and promotes pulmonary vascular remodeling. These two points help to increase pulmonary vascular resistance, which leads to the aggravation of PH in Kcnk3-mutated rats and the subsequent RV hypertrophy and dysfunction. Of note, potassium channel expression is regulated by BMP signaling (61). This suggests that BMPR2 mutations may also play a role in PH-LHD. Another study showed that a missense variant (rs1799983) in the endothelial NOS (NOS3) gene has been related to pulmonary vascular remodeling in CpcPH (62).

In addition, research on the epigenetic dysregulation of genes has made breakthroughs in recent years. François Potus and other scholars (63) believed that the main modifications of epigenetic mechanisms include DNA methylation, histone acetylation, and micro-RNA production. DNA methylation is a good epigenetic modification. In addition, the pathological activation of DNA methyltransferase could increase DNA methylation of specific genes associated with disease progression (64). Despite interactions between methylation sites and methyl-binding proteins, excessive methylation usually inhibits gene transcription, which will change the expression of other genes (65). The translocation of methylcytosine dioxygenase 2 (TET2) is a common gene mutation (66), and it is a key regulator for DNA demethylation. It can catalyze the transformation of methylated nucleotide 5-methylcytosine into 5-hydroxymethylcytosine. TET2 contributes to the epigenetic regulation of gene expression by removing methyl groups on DNA and binding to histone deacetylase. The loss of TET2 function will increase DNA methylation and lead to pulmonary vascular remodeling (67, 68).

### Inflammatory cells and inflammatory factors

Notably, pulmonary vascular remodeling not only indicates passive congestion but also involves active remodeling of the pulmonary vasculature. Cumulative evidence suggests that inflammation generally modulates disease progression comprising key pathogenic drivers of vascular remodeling in PH-LHD (69). Inflammation can mediate pulmonary vascular remodeling, including the complex interaction between body fluids and cytokines triggered by infectious, toxic, and autoimmune diseases. Inflammatory cells include mast cells, T lymphocytes, B lymphocytes, neutrophils, macrophages, and ECs. These inflammatory cells are capable of releasing lipid mediators, chemokines and cytokines that further promote the recruitment of immune cells, leading to the manifestation of an inflammatory state (1). Taking mast cell as an example, it is a tissue cell of the body involved in immune regulation, and its structural feature includes a large number of basophilic particles in its cells (70). Regarding the accumulation of mast cells, Fernández et al. found that mast cells could release a wide range of mediators (21), including vasoconstrictive proliferative serotonin; histamine; and cytokines such as IL-6, protease, chymase, and trypsin. In addition, mast cell granules stimulate EC proliferation once released (50). Therefore, activated mast cell can promote the proliferation of smooth muscle cells. Based on the potential role of mast cells in pulmonary vascular remodeling of PH-LHD, mast cell accumulation is another mechanism of vascular remodeling (50). Meanwhile, some investigators have suggested that macrophage expansion and monocyte recruitment are key pathogenic drivers of vascular remodeling. Monocytes can actively recruit to the site of injury in response to injury and pathological stimuli, and under prolonged activation of EC, EC upregulate the expression of adhesion molecules while secreting growth factors and cytokines, which in turn affect SMC proliferation and apoptosis, facilitating transcytosis migration of inflammatory cells, leading to vascular remodeling. This also confirms that factors regulating endothelial cell function contribute to pulmonary vascular remodeling. Activated EC can also release inflammatory factors such as GM-CSF, CCL2, and IL-6 to promote leukocyte recruitment and accumulation. Accumulated leukocytes release other factors, which in turn induce a pro-inflammatory phenotype of EC and further recruitment of inflammatory cells (71). Many cytokines have also been found to regulate dysfunction under pulmonary hypertension. For example, in one study, Villar-Fincheira et al. (72) identified IL-6 as a multifunctional pro-inflammatory cytokine and found a role in pulmonary vascular remodeling, which can induce PASMC proliferation through activation of stat3 (73). Other studies demonstrated the involvement of IL-6 in the formation of PH-LHD, which can be used to diagnose pulmonary hypertension associated with left heart disease and assess the severity of the disease (74). Another experimental study showed that (75) the defect of BMPR2 signaling could also induce pulmonary artery smooth muscle cells to produce inflammatory cytokines, thereby aggravating the inflammatory response and promoting the development of pulmonary hypertension. However, whether the specific mechanism of BMPR2 causes imbalance and pro-inflammatory effect or whether these two factors are mutually enhanced in the vicious circle of PAR remains unclear. Therefore, the etiology and pathogenesis of inflammatory cytokines must be further studied.

### Mitochondrial dysfunction

In eukaryotic cells, mitochondria are key organelles that mediate complex and integrated metabolic pathways in bioenergetics, biosynthetic pathways and cell signaling. There are seven main metabolic pathways involved in mitochondrial function related to PH-LHD, including glucose and fatty acid oxidation, glutamine catabolism, arginine metabolism, a carbon metabolism, reduction and oxidation (REDOX) reactions, and the tricarboxylic acid (TCA) cycle and electron transport chain (ETC) (76). An example is mitochondrial dysfunction in PASMC: the metabolic transition from glucose oxidation to uncoupled aerobic glycolysis. In the presence of oxygen, the rate of glycolysis in normal vascular cells is closely related to the rate of glucose oxidation. In Warburg metabolism, uncoupled glycolysis is increased because glucose oxidation is inhibited and glycolysis is disproportionately elevated, providing enough energy for abnormal cells to thrive (77). Freund-Michel et al. (78) believed that mitochondrial dysfunction is involved in the pathogenesis of PH-LHD, playing a central role in the metabolic theory of this disease. In pulmonary vascular cells, the metabolic shift toward glycolysis increases the availability of non-oxidized amino acids, lipids, and sugars, which are all necessary for rapid cell proliferation. It can slow down apoptosis and cause excessive cell proliferation and vascular remodeling. During aerobic glycolysis, pulmonary vascular cells showed increased mitochondrial reserve respiratory capacity, which depends on increased fatty acid oxidation and is associated with hemodynamic changes in PH-LHD (79). Therefore, the disorder of mitochondrial metabolism is a key feature of PH-LHD pathology.

**Figure 6:**
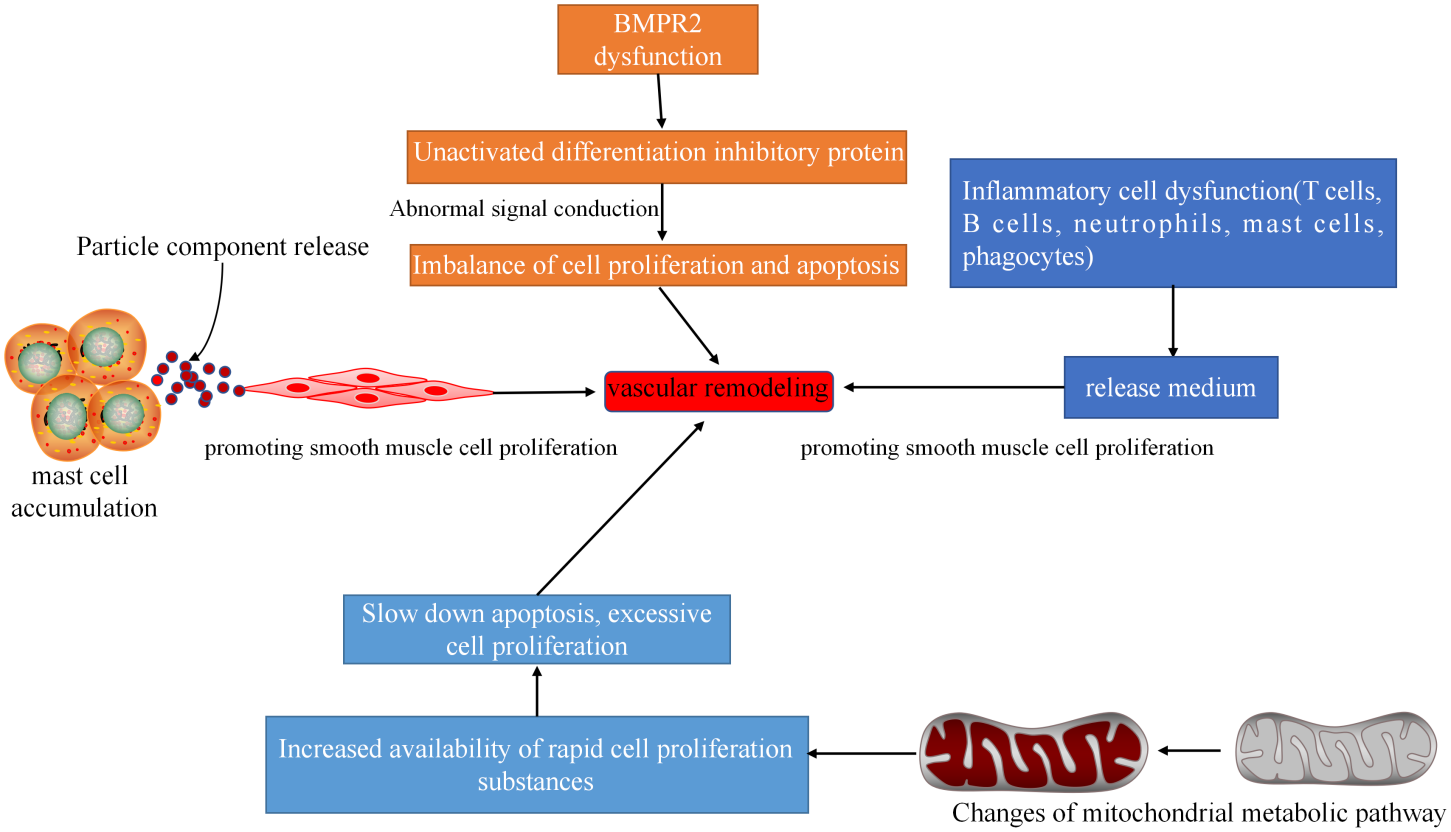
Related pathways of vascular remodeling. Inflammatory cell dysfunction can produce dysfunctional inflammatory cytokines, mediate cell proliferation, and cause vascular remodeling. Dysfunction of BMPR2 will result in the inability of the differentiation protein inhibitor to be activated, and the signal cannot be transmitted, resulting in imbalance of cell proliferation and apoptosis and vascular remodeling. A large number of accumulated mast cells can release media, promote the proliferation of smooth muscle cells, and cause vascular remodeling. Changes in mitochondrial metabolic pathways can increase the availability of rapidly proliferating substances in cells, thereby slowing apoptosis and causing excessive cell proliferation and vascular remodeling.

Vascular remodeling is a physiological adaptive change made by the human body to adapt to the increase in filling pressure of the left ventricle caused by left heart disease. The thickening of the vascular wall and the decrease of the lumen diameter simultaneously lead to or aggravate pulmonary hypertension. The mechanisms involved include genetic inheritance, ion channels, mast cell accumulation, inflammatory factors, and mitochondrial dysfunction (Figure 6). These aspects should be considered to study vascular remodeling.

### Notch signaling pathway

Notch signaling is a key regulator of vascular morphology and function and plays a critical role in the regulation of VSMC phenotypic transition. Among them, Notch3 is basically expressed only in VSMCs, which can control the proliferation of VSMCs and maintain the smooth muscle cells in an undifferentiated state (80). Under pathological conditions, Notch3 primarily regulates the VSMC transition between contractile and synthetic phenotypes, and aberrantly expressed Notch3 signaling plays an important role in vascular remodeling (81). Li. et al. found through experimental and investigational studies that the expression of Notch3 signaling pathway components was increased in pulmonary vascular smooth muscle cells of patients with PH-LHD and that disease severity correlated with the amount of Notch3 protein expressed in human and rodent lungs (82). Notch protein is a cell membrane receptor that mediates intercellular signaling, and its ligands are mainly expressed in endothelial cells, and Notch signaling is transmitted through cell–cell interactions (80). Because of the important role of Notch3 signaling in vascular function and its specific expression pattern in VSMC, it can be included as a candidate therapeutic target for PH-LHD disease. However, many of its specific signaling pathways and molecular mechanisms remain unknown due to the paucity of information on the potential mechanistic role of Notch3 in PH-LHD and vascular remodeling.

### Multiple factors affect vasoconstriction and vascular remodeling, leading to PAR and PH-LHD

Three pathways in vasoconstriction are also associated with vascular remodeling, such as prostacyclin, ET-1 with calcium channels, and NO with potassium channels, which can lead to the contraction and proliferation of PASMCs, which trigger PAR and PH-LHD (83). Many potassium channels are found in PASMCs, and the latter two of the abovementioned pathways are related to potassium channels. Therefore, studying the potassium channels is necessary to reveal the pathogenesis of PH-LHD. The functional expression of potassium channels in PASMCs and pulmonary artery endothelial cells (PAEC) is manifested in three different levels (1, 84) Regulation of membrane potential in PASMCs to control pulmonary vascular tension; (2) Regulation of PASMCs by controlling apoptosis, survival, and proliferation; (3) Maintaining resting membrane potential and calcium entry in PAEC to regulate the release of endothelial vasodilators. Increased intracellular calcium concentration is the initiating factor of vascular contraction and smooth muscle cell proliferation (85). Normal PASMCs need a proper balance between proliferation and apoptosis, and imbalance of these two mechanisms will lead to pulmonary vascular remodeling. Jin et al. (86) found that the inhibition of miR-206 could increase the expression of voltage-dependent potassium channel current in PASMC, thereby slowing the progression of PH, also in PH-LHD patients, they found a significant reduction in miR-206 levels, which correlated with increased PASP. Therefore, miR-206 can be used as an important predictor for PH-LHD.

## Treatments

Compared with the latest progress of PAH treatment, PH-LHD treatment has not made significant progress. In this case, the main strategy for treating PH-LHD, according to currently published guidelines, is to treat primary left heart disease. These include controlling risk factors for cardiovascular disease, drug therapy (including diuretics, angiotensin converting enzyme inhibitors, beta blockers, etc.), interventional therapy (valve repair, coronary reperfusion therapy, ventricular resynchronization therapy, left ventricular assist device, heart transplantation, etc.) and treatment of complications (COPD, sleep apnea syndrome, pulmonary embolism, etc.). The difference between PAH and PH-LHD treatment strategies is that PH-LHD requires optimal treatment of primary left heart disease before considering the treatment of PH. In clinical practice, PH-LHD patients are often mistakenly classified as PAH and treated with PAH-specific drugs. According to the 2022 ESC/ERS Guidelines for the diagnosis and treatment of pulmonary hypertension, drugs approved for PAH are not recommended in PH-LHD. Targeted drugs approved for the treatment of PAH include endothelin receptor antagonists (ERA), prostacyclin analogs, phosphodiesterase-5 inhibitors (PDE 5i) and soluble guanylate cyclase (sGC) stimulators (87, 88). Endothelin receptor antagonist can block endothelin, which is an effective vasoconstrictor and can also stimulate cell proliferation and thrombosis. A number of trials of endothelin receptor antagonists in the treatment of HFrEF have been conducted, and the results have shown no improvement or deterioration in edema and treatment (89–91). PDE 5i have beneficial acute hemodynamic benefits, including improved gas exchange, skeletal muscle function, diastolic function and RV function in PH-LHD patients (92). Sildenafil is a PDE 5i that was originally developed for the treatment of systemic hypertension and angina. One study examined the acute hemodynamic effects of sildenafil, nitric oxide and both in 11 patients with dilated cardiomyopathy and PH. Studies have shown that the average PA and PVR decreased significantly (93). The sGC stimulator riociguat (which is approved for PAH) was investigated in two randomized controlled trials in PH-LHD. In patients with PH-HFrEF, the LEPHT trial failed to reach the primary endpoint of lowering PAP vs. placebo, but led to a substantial increase in cardiac index, resulting in lowering of PVR (94). Similar results were obtained in another experiment (95). Patients with PH-HFrEF were treated with intravenous epoprostol in a trial that was terminated early because of a tendency to increase mortality in the treatment group compared to the placebo group (96). So far, no large-sample randomized controlled clinical trials have confirmed that targeted drugs can benefit PH-LHD patients (97). In clinical treatment, it is particularly important to distinguish IpcPH and CpcPH, and their treatment strategies are not exactly the same. Individualized treatment is recommended for LHD and CpcPH patients with severe precapillary components. In future clinical trials, strict inclusion criteria should be established to distinguish between IpcPH and CpcPH and to screen out PH-LHD patients who may be sensitive to targeted drugs.

## Conclusion

Currently available pharmacological therapies for PH-LHD (including diuretics, angiotensin-converting enzyme inhibitors, and beta-blockers) have improved the outcome of PH-LHD, but the disease remains progressive and ultimately fatal. To date, no large sample of randomized controlled clinical trials has confirmed that targeted drugs can benefit patients with PH-LHD. In addition, safety considerations of therapeutic agents should not be overlooked. Therefore, determining targeted drugs suitable for such diseases through continuous research on the etiological mechanisms is important.

As previously mentioned, experimental and clinical studies have shown that inflammatory factors are a concept in the pathology of PH-LHD vascular remodeling, emphasizing the influence of inflammation on the progression of PH-LHD and as a prognostic marker to predict the outcome of the disease more accurately than hemodynamic parameters. At present, the study of novel targeted drugs in the direction of inflammatory factors is a new approach to improve the treatment of pulmonary hypertension caused by left heart disease. In addition, relevant literature points out the importance of an intact immune system for the development of PH-LHD. Given the effect of T and B cells of the immune system on the condition of PH, therapeutic advances in PH-LHD could open a novel avenue for PH-LHD treatment by negatively modulating T cell immune response.

The pathogenesis of PH-LHD studied at home and abroad primarily revolves around vasoconstriction and vascular remodeling, but the exact target remains unclear. We have summarized two target directions through extensive literature reading, namely, ion channels (including ET-1 with calcium and potassium channels, prostacyclin, and NO with potassium channels) and mitochondria, and other influencing factors produce a series of physiopathological responses by acting on these two targets. However, the exact target action pathway still needs to be further investigated. We also summarize the abovementioned research articles on the pathogenesis of PH-LHD at home and abroad to provide reference for the research on the mechanism of PH-LHD and clinical treatment, particularly molecular targeted therapy.

## Author contributions

MX and DL wrote the manuscript, figure legends, and created the figures. YY, QW, and CZ revised the manuscript. All authors contributed to the article and approved the submitted version.

## Funding

This work was supported by the National Natural Science Foundation of China (8217020711) and Guangdong Provincial Science and Technology Department Rural Commissioner Special (No. KTP20200165).

## Conflict of interest

The authors declare that the research was conducted in the absence of any commercial or financial relationships that could be construed as a potential conflict of interest.

## Publisher’s note

All claims expressed in this article are solely those of the authors and do not necessarily represent those of their affiliated organizations, or those of the publisher, the editors and the reviewers. Any product that may be evaluated in this article, or claim that may be made by its manufacturer, is not guaranteed or endorsed by the publisher.
